# Modelling the upwelling radiance detected in a seawater column for oil-in-water emulsion tracking

**DOI:** 10.1038/s41598-023-50570-x

**Published:** 2023-12-28

**Authors:** Barbara Lednicka, Zbigniew Otremba, Jacek Piskozub

**Affiliations:** 1 Department of Physics, Faculty of Marine Engineering, Gdynia Maritime University, 81-225 Gdynia, Poland; 2 Institute of Oceanology Polish Academy of Sciences, 81-712 Sopot, Poland

**Keywords:** Environmental sciences, Ocean sciences

## Abstract

This study concerns changes in the solar light transfer in seawater as a result of the appearance of oil substances in the form of oil-in-water emulsions. The expected effect of the studies is to gather knowledge that would be useful in designing an optical sensor for monitoring oil substances penetrating the seawater column. The paper presents the process of the Monte Carlo modelling of the upwelling radiance detected by a virtual underwater sensor. Moreover, this article discusses the predicted difference between the intensity of the signal from the upwelling radiance meter and the signal from the downwelling radiance meter. The modelling results suggest that in order to obtain a strong signal indicating the presence of oil substances, the radiance meter should receive light running upwards in the sea depth.

## Introduction

Oil and natural gas account for over 90% of the value of all mineral resources currently extracted from the seabed^1^. Spills of crude oil into the oceans and seas are a significant ecological threat to marine ecosystems and result mainly from anthropogenic activities. Possible causes of oil spills into marine waters include maritime transport, oil platforms, ships sinking, shipwrecks on the seabed or damage to pipelines^2^. Direct damage caused by a major oil spill (such as those mentioned above) is always easy to detect and assess. In contrast, the effects of small but continuous oil spills that occur in every port are much more difficult to quantify because they create persistent pollutants that natural processes cannot break down. This slow settling of oil on the seabed can lead to long-term contamination of the seawater column and sediments. Oil spills in coastal waters concern areas that are valuable for the species of fauna and flora found there^1^.

The detection of oil in the marine environment becomes difficult if it takes a dispersed form and penetrates deep into the water. Parsa et al.^3^ investigated vertical oil dispersion of surface oil spills under non-breaking regular waves. They studied the variation in oil concentration caused by oil dispersion in a water column and found that the oil concentrations near the flume bed were 12–33% of the concentration near the water surface, and the concentrations at mid-depth were 44–77% of the surface concentrations at the surface^3^. Oil spills can contaminate the water column in several ways and from various sources. Heavy oils from a surface spill can either become submerged oil while still in the water column or sunken oil when it reaches the sea bottom. Sometimes, oil may also rise to the surface from the seabed outflow. In addition, small oil droplets can result from applying chemical dispersants to a surface spill or natural dispersion, or due to the seabed outflow^4^. Oil sampling using net snares towed through the water column is time-consuming and laborious and provides only general information about the amount of oil, depth and geographical location. Therefore, attempts are being made to create remote oil spill monitoring systems based on optical or acoustic sensors^4^.

The ecological disaster in 2010 caused by an oil spill from the Deepwater Horizon in the Gulf of Mexico revealed several technological deficiencies in responding to oil spill circumstances. The lack of quick and efficient technology for monitoring and sampling the water column to determine the extent of oil pollution contributed to the scale of this tragedy. Therefore, there is an urgent need for monitoring technology for a streak of oil suspended in a water column which provides data in real-time^4^.

It is believed that by researching water-light interactions, an innovative method for monitoring water resources (inland waters and coastal areas) can be developed, which will enable oil contamination control. This issue will make it possible to expand the necessary knowledge for environmental management to improve the ecological status of coastal ecosystems. The potential of the Apparent (and Inherent) Optical Properties (AOPs and IOPs) models of waters (for example, ocean color algorithms) has been confirmed by recent data sets and algorithms, but many of their aspects are still not well understood^5^. The most important aspect, which requires more research efforts, is associated with coastal areas as the related flows of organic matter in the coastal ocean are disproportionately higher in comparison with open ocean regions^6,7^. There is great potential in water monitoring, which can help to establish trends in biogeochemical parameters of semi-enclosed seas, coastal areas, rivers and inland water areas^8–11^, i.e. areas classified as Case 2 waters^12^. The innovative models developed are useful in improving the ecological status of these water areas. Moreover, the application of monitoring in rivers and coastal waters allows precise control of the biogeochemical water components, which are highly responsible for reducing marine health.

Coastal areas and semi-enclosed seas are also at risk of oil spills due to sea level rise. It is also important for these areas to identify how climate change affects offshore extraction (oil and gas) and the associated oil spill risk^13^. Rising sea levels can significantly increase the likelihood of erosion and damage to oil and gas infrastructure. It is crucial that appropriate measures are taken to mitigate and, above all, prevent the negative impacts of oil spills. Therefore, it should be observed in what way water contamination, covering different types of suspended and dissolved matter, oil pollution, etc., determines the penetration of light in shallow waters^14^. Coastal areas are highly susceptible to sea level rise, changes in the frequency and intensity of storms, increasing precipitation, and warmer ocean temperatures. It is estimated that throughout the twentieth century, the global sea level rose by (1.7 + /- 0.5) mm/year^15^. Shoreline erosion, coastal flooding, and water pollution affect man-made infrastructure and coastal and river ecosystems. Moreover, semi-enclosed seas, coastal areas and rivers are among the richest biological regions of ocean ecosystems and are also more exposed to harsh environments. These areas are particularly exposed to anthropogenic organic contamination. Deterioration of them can have severe economic and social consequences, stressing the necessity for developing new monitoring tools for biochemical contamination. The wide range of human activities that take place in coastal areas highlight their economic and social importance but also indicate the multiple potential sources of pollution^16^. Therefore, there is an urgent need to design a unique tool for observations of water quality and especially of any kind of pollution to protect these water ecosystems.

The Baltic Sea coastal area is inhabited by one hundred million people and has obvious social, economic and ecological significance. The coastal waters in the study region are characterized by a higher load of organic and inorganic particles originating especially from river runoff and higher concentrations of colored dissolved organic matter (CDOM) than the open sea waters^17^. Hieronymi et al.^18^ showed the considerable impact of the CDOM absorption on remote sensing reflectance in the Baltic Sea waters. Accordingly, regional features such as the high values of IOPs and AOPs should be taken into account in the modelling. Oil spills are a very crucial problem in coastal waters, especially in port waters. The studies of the upwelling radiance as proposed here can be used as indicators of water quality for these areas. Therefore, the southern Baltic Sea was selected as the research field.

The components of seawater strongly influence its optical properties. Natural reservoirs are centers which are more or less rich in dissolved and suspended matter. Due to the large temporal and spatial variability of these water components, the light absorption and scattering coefficients are also characterized by significant fluctuations, and their values rarely resemble those of pure water. The values of absorption and scattering coefficients measured in optically different water bodies may differ by up to several orders of magnitude. Type one waters, i.e. open ocean waters with very low concentrations of suspended and dissolved matter, in which the light absorption coefficient is dominated by phytoplankton, are usually blue. In the coastal zone, i.e. in Case 2 waters, there is a high concentration of optically active components, and the light absorption coefficient is dominated by seawater components (especially dissolved organic substances). The values of the scattering and absorption coefficients depend on the concentration and chemical composition of optically active admixtures found in seawater. With regard to the backscattering coefficient, this is clearly visible in the plots of scattering phase functions measured by Petzold^19^ for three different types of water bodies characterized by different proportions of suspended and dissolved matter. This is because the interpretation of, for example, the upwelling radiance of these waters is not clear, and the algorithms based on it for estimating various biotic and abiotic parameters of the sea are much more complex and can only be used locally. Therefore, the current method could be used only locally for the Southern Baltic area.

Petroleum substances in the form of oil-in-water emulsions disperse in seawater, thus contributing to the turbidity of water masses. Turbidity is a conventional value indicating the presence of various types of suspensions in water, referred to as Suspended Particulate Matter (SPM). SPM is also referred to as Total Suspended Solids (TSS), Total Suspended Matter (TSM), and Total Particulate Matter (TPM)^20^. Stramski^21^ suggests that the substances accounting for turbidity include organic substances, i.e. autotrophic and heterotrophic plankton, bacteria, viruses, and detritus, as well as mineral particles. In oceanology, a number of correlations have been shown between turbidity and specific features of water masses^16,22^. The most convenient method for determining turbidity is the optical method, based on light scattering, where Formazine suspension is used as a standard. The unit of turbidity measured in this way is the Formazine Nephelometric Unit (FNU). Then turbidity can be used as a proxy in determining, e.g. SPM^23^. Water turbidity meters may react to the presence of an oily suspension, but one can never be sure that the changes in the reading are caused by the oil. The spread of light in the water column and its characteristics determined by spatial, directional and spectral distribution of radiance can be tracked, provided that the absorption coefficients, scattering coefficients and angular scattering functions of all water components are known. Such an approach was reported in our previous work^24^, where a spectral index of 555/412 was identified as suitable for the underwater detection of oil-in-water emulsions, with the reservation that this applies to the southern Baltic Sea under cloudless sky conditions. Strictly the same approach, model and methodology were used in the research presented in this paper. This also applies to the state of the sea surface and the optical model of the sky. The only difference is the virtual photon receiver. It was rotated by 180 degrees (directed downwards) so that it registered photons running from the water column perpendicularly upwards. A virtual radiance meter (photon receiver) counted photons traveling upwards to an 8-degree cone.

In this paper, only the vertical position of the radiometer was considered. For underwater radiometry applications, the radiance sensors should be deployed vertically. During wave-tilting or current-tilting of the measuring buoy, the non-verticality of radiometers will result in uncertainty in the measurements of downwelling irradiance E_d_(z) and upwelling radiance L_u_(z) because of the anisotropic nature of the downwelling and upwelling light fields^25^.

In 2020, Tilstone et al.^26^ made attempts to estimate the differences in the accuracy of above-water radiometer measurements used for the validation of ocean color products. They compared ten measurement systems. As a baseline reference for comparisons, they used the weighted average of five above-water measurements. Their studies showed that E_d_ accounted for the largest fraction of the variance in remote-sensing reflectance (R_rs_). Moreover, in the case of long-term under-water measurements, a change in the transmissivity of the optics in the radiometer may occur, e.g. due to the growth of algal films, or in the case of radiometers that measure downwelling radiance by settling of sediments on the instrument window. In view of the above, radiometers that measure L_u_ appear to be more accurate and less susceptible to contamination than radiometers that measure downwelling radiance (L_d_)^27^. Therefore, we assume that, for the purposes of underwater monitoring, better results will be obtained by using a radiometer that measures upwelling radiance than radiometers that measure downwelling radiance. Moreover, in 2013, D’Alimonte et al.^28^ conducted a study on, among others, the regression of in-water radiometric profile data with the objective of investigating solutions to minimize uncertainties of derived products (upwelling radiance and downwelling irradiance). For this purpose, they used Monte Carlo simulations to generate radiometric profiles. This work is a good example of the importance of virtual environments to assist the analysis of field data and processing methods. The main goal of their research was to estimate the uncertainties of regression data products in the presence of light field perturbations due to surface waves. Their algorithm produced simulated radiometric profile data characterized by deep water and vertically homogeneous IOPs. Therefore, it was decided not to extend the simulation for additional variables such as variability of the IOPs of oil-free water across multiple sea areas, sun heights, weather conditions and various oils. However, the study focused on the major question of determining which radiometer setting for oil pollution monitoring would be the most effective. The main objective of the work was to estimate which of the two measuring methods (described in the current paper and in^24^) allows for better detection and characterization of oil that is entrained and dispersed in the water column.

## Materials and methods

**Figure 1:**
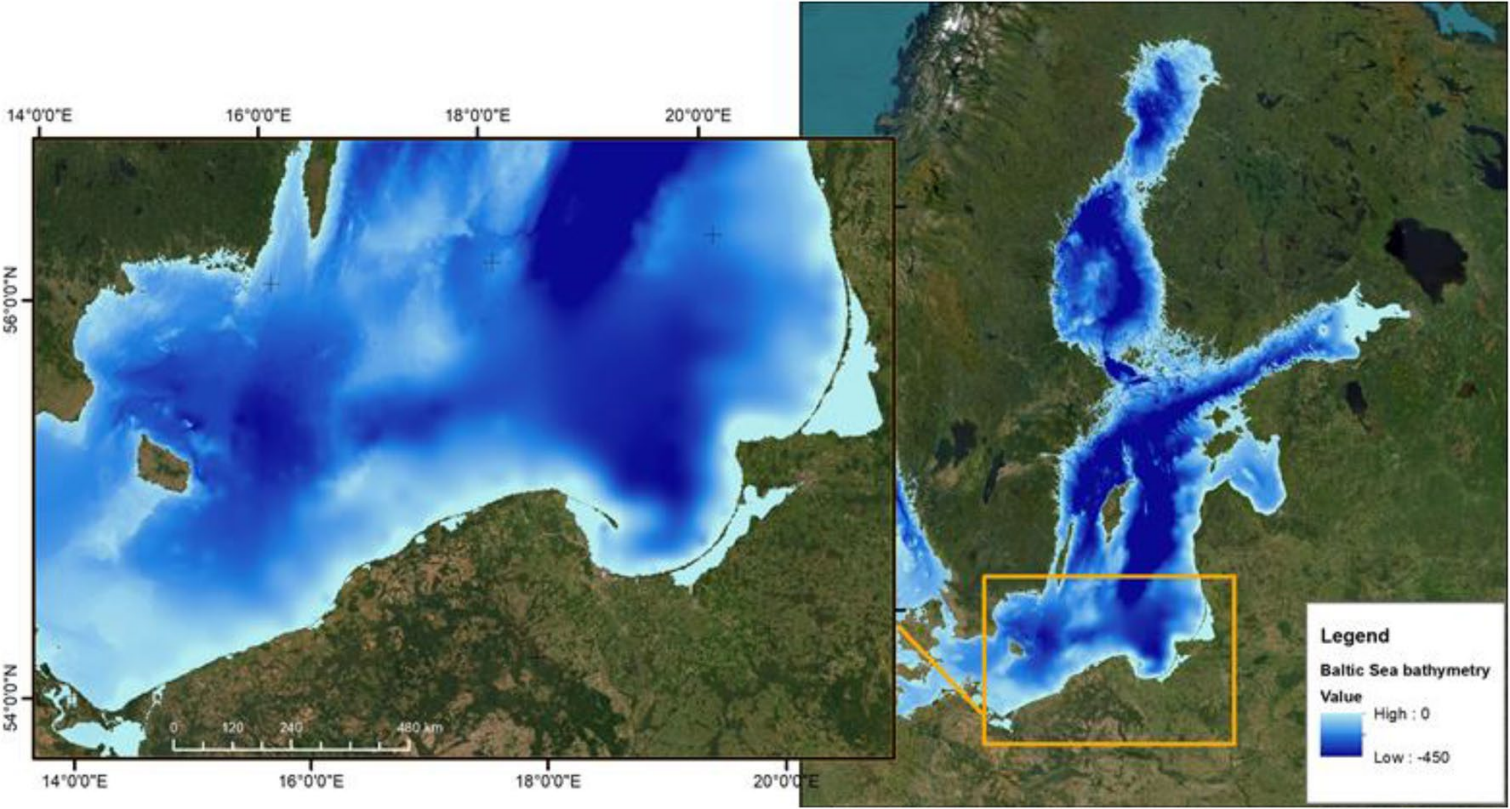
Location of the study area for which an optical model of the sea was made (ArcGIS Desktop 10.7.11595 https://www.esri.com/en-us/arcgis/products/arcgis-desktop/overview).

To check if the virtual facing downwards radiance meter (measuring upwelling radiance) is a suitable tool for detecting oil in the sea column, in situ data of selected optical properties was analyzed. The measurements of the absorption and the scattering coefficients were carried out by Sagan^29^ for several years in waters of the southern Baltic (Poland) (Table 1), the area from which values of the IOPs were used to model light propagation in seawater (Fig. 1).

**Table 1:** The values of total absorption (*a*_*t*_(λ)) (absorption by dissolved and suspended particles and water molecules) and total scattering (*b*_*t*_(λ)) (scattering by suspended particles and water molecules) coefficients for the eight wavelengths, based on^29,30^.

Wavelength (*λ*) [nm]	412	440	488	510	532	555	650	676
Absorption Coefficient (*a*_*t*_) [m^**−**1^] 0–5 m	0.596	0.398	0.218	0.188	0.163	0.149	0.391	0.517
Absorption Coefficient (*a*_*t*_) [m^**−**1^] 5–10 m	0.536	0.348	0.178	0.158	0.143	0.139	0.381	0.497
Scattering Coefficient (*b*_*t*_) [m^**−**1^] 0–5 m	0.63	0.60	0.60	0.60	0.60	0.59	0.54	0.51
Scattering Coefficient (*b*_*t*_) [m^**−**1^] 5–10 m	0.39	0.37	0.37	0.37	0.37	0.37	0.34	0.32

The values of optical properties at an oil concentration of 10 ppm in water used in the sea environment optical model are shown in Table 2.

**Table 2:** The values of absorption (*a*_*oil*_(λ)) and scattering (*b*_*oil*_(λ)) coefficients of oil-in-water emulsions (with a concentration of 10 ppm) for the eight wavelengths, based on^31,32^.

Wavelength [nm]	412	440	488	510	532	555	650	676
Absorption coefficient (*a*_*oil*_) [m^**−**1^]	0.299	0.114	0.052	0.042	0.029	0.029	0.0125	0.0087
Scattering coefficient (*b*_*oil*_) [m^**−**1^]	7.81	7.97	7.98	7.95	7.91	7.87	7.60	7.48

In this paper, Monte Carlo simulations were used to model the radiance below the southern Baltic Sea surface based on known values of IOPs and oil-in-water emulsions for the eight wavelengths (Tables 1, 2). Our computational algorithm relied on simulating the “life path” of a huge number of virtual photons (two billion virtual photons). This method is often used to model the light field (angular distribution of radiance) in the water column^31,32^. Ansi C was used for Monte Carlo modeling. This code has already been used in the authors previous studies^24,31,32^ to describe the light field in seawater. This method involves counting virtual photons in oil-free and oil-polluted marine water. Both oil-polluted and oil-free waters have assigned absorption and scattering values with different scattering phase functions^19^. The values of total absorption (absorption by dissolved and suspended particles and water molecules) and the scattering coefficients (scattering by suspended particles and water molecules) of the Baltic waters were used to model light propagation in seawater (Table 1). The values of absorption by dissolved and suspended particles, according to Sagan^29^, are based on the results of in situ measurements from 1999 and 2003–2005. The values of absorption and scattering coefficients of seawater molecules were taken from Buiteveld^30^. The absorption and scattering properties of crude oil extracted from the Baltic Sea were also utilized, which can exist as an oil-in-water emulsion with a concentration of 10 ppm^31,32^ (Table 2). Based on the chosen phase functions and scattering and absorption values (using pseudo-random numbers, scattering angles in 3D and probability distributions of the pathways), photons were traced in a stochastic way. This allowed to the recording of bidirectional reflectance distribution functions (BRDF) and radiance and irradiance values.

**Figure 2:**
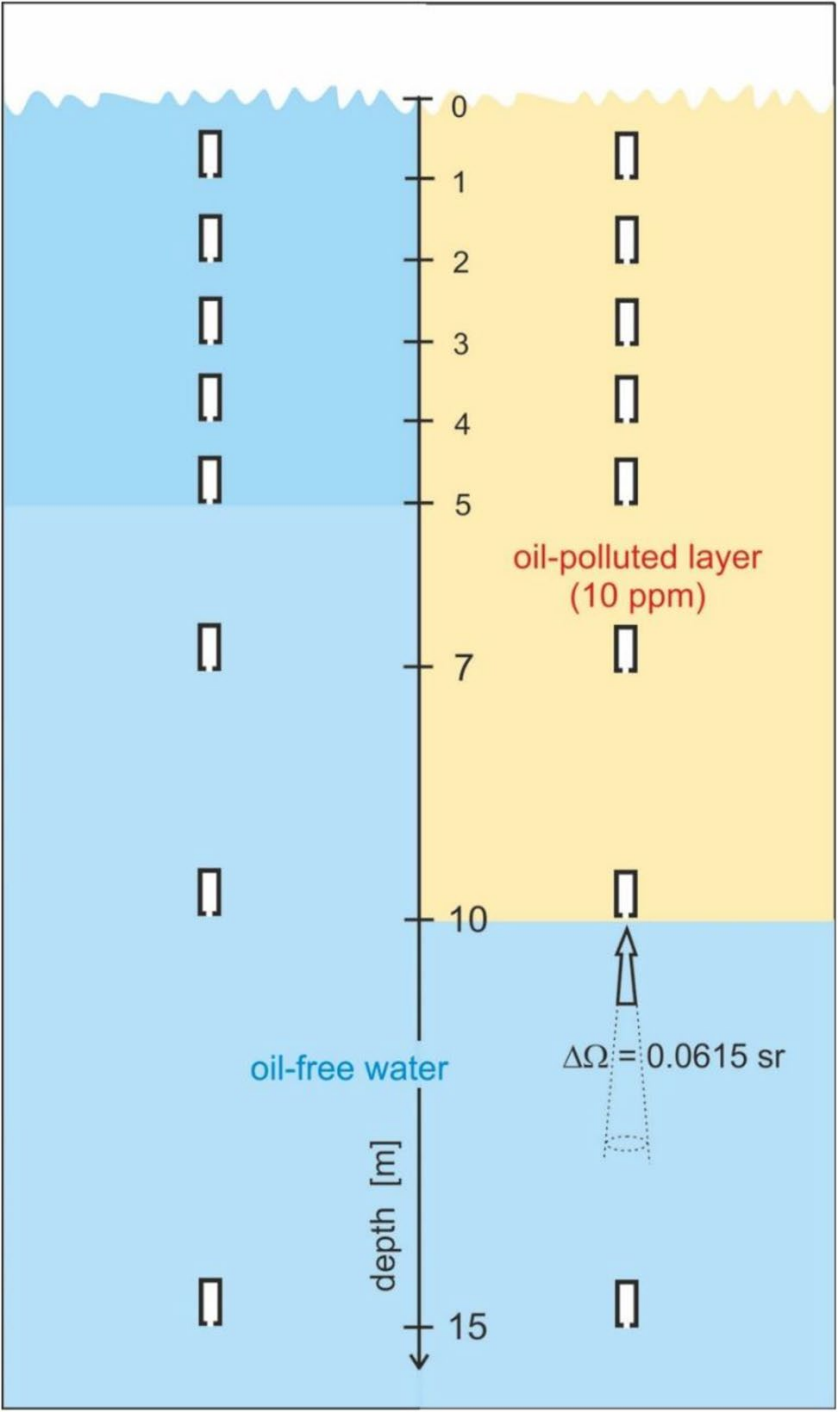
The model of the sea with the placement of virtual upwelling radiance meters and their geometry.

The state of the sea surface and the optical model of the sky were based on the Cox and Munk^33^ model, where the wind speed is 5 m/s, and as a result, the water surface is virtually wavy. The virtual photon receiver was directed downwards so that it registered photons moving from the water column perpendicularly upwards (Fig. 2).

**Figure 3:**
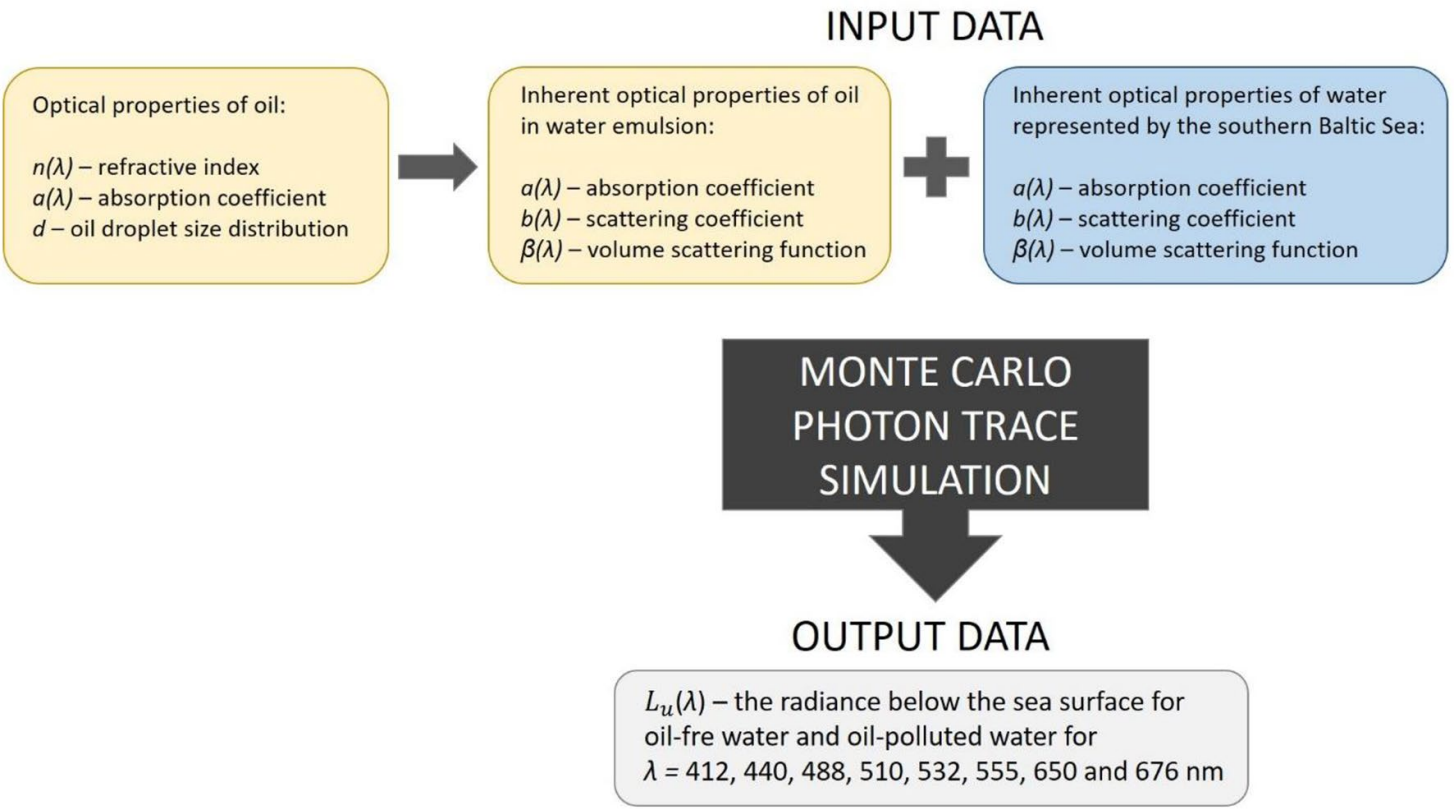
Block diagram of the model of the radiance below the southern Baltic Sea surface.

The radiance is usually measured by limiting the field-of-view (FOV) of the radiometer and assuming radiance is spatially invariant or changes little over the projected solid angle. It is assumed that the in-water full-angle FOV for the upwelling radiance bands should be smaller than 20°^34^. The radiance sensors generally measured the nominal full angle field-of-view (FOV) in the range of 6°–8.5°^26,35^. Therefore, the virtual radiance meter counted photons traveling upwards from an 8-degree cone. Moreover, it was assumed in the simulation that the polluted water layer is 10 m thick (Fig. 2). Figure 3 presents a block diagram of the model of the radiance below the southern Baltic Sea surface.

To estimate the contribution of light coming directly from the sun and light coming from the upper hemisphere of the sky (for different wavelengths), the values of direct solar irradiance percentage obtained by Greg and Carder^36^ from an irradiance model (Table 3) were used. This algorithm computes irradiance at or just below the ocean surface at high spectral resolution in the range of 350–700 nm.

**Table 3:** The direct solar irradiance percentage for the eight wavelengths, based on^36^.

Wavelength [nm]	412	440	488	510	532	555	650	676
Direct solar irradiance percentage	64.7	66.2	68.2	68.8	69.4	69.9	71.2	71.4

## Results and discussion

**Figure 4:**
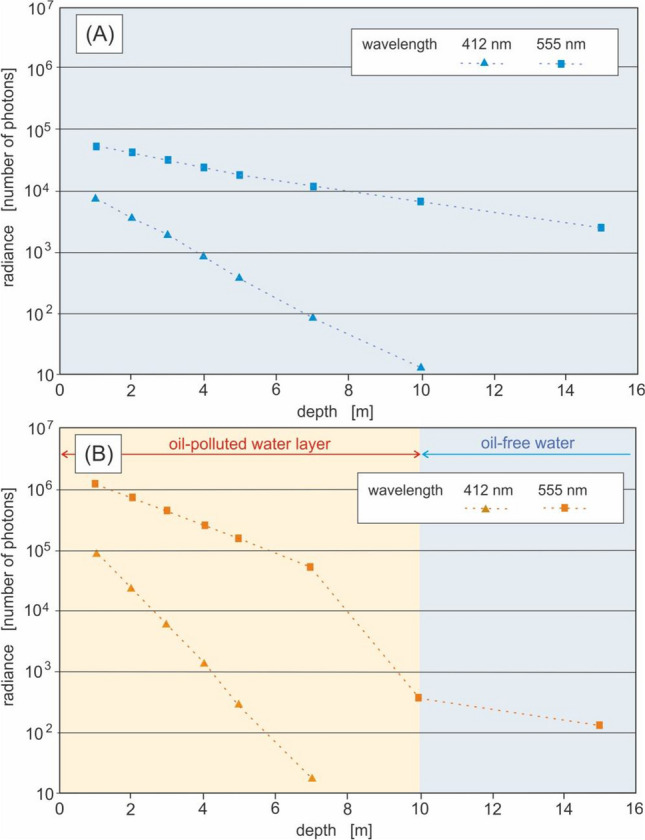
The values of upwelling radiance for chosen wavelengths under the sea surface for oil-free seawater (**A**) and oil-polluted water (**B**).

Radiance meter readings were simulated at various depths for eight light wavelengths (412, 440, 488, 510, 532, 555, 650 and 676 nm). It has been established based on previous studies^24,37,38^ that the spectral index of 555/412 is the most favourable combination of two wavelengths for the underwater detection of oil-in-water emulsions. Therefore, Fig. 4 presents the values of upwelling radiance for these two wavelengths under the sea surface for oil-free seawater (Fig. 4A) and oil-polluted water (Fig. 4B).

Figure 4 shows significant differences between the values of radiance for both unpolluted and polluted seawater. The value of upwelling radiance recorded within the first few meters of polluted water is higher than for clean water. However, the deeper the detector is immersed, the more similar the radiance values become. At greater depths, depending on the wavelength, the radiance in clean water exceeds that in polluted water. Such an effect is a consequence of the sum of photon absorption and scattering events. The probability of photon absorption along a certain path of its travel in water is related to the absorption coefficient, while the probability of scattering depends on the scattering coefficient. A photon is scattered in a random direction, and this effect is represented by the phase function associated with water and individual water components. At a depth of the first few meters below the sea surface, the value of upwelling radiance in water contaminated with an oil emulsion is higher than in clean water. This phenomenon should be explained by the predominance of scattering events over absorption. But at a depth of several meters, the radiance in polluted water is lower than that in clean water. A radiance meter installed in a polluted sea, at the boundary between the polluted layer and the clean water layer, does not record the polluted water properties. Nevertheless, it shows lower radiance values than the ones that would be recorded in oil-free water. There are probably two reasons for this, namely the reduced amount of light under the oil emulsion layer and the weaker light scattering in the clear water zone. In the strongly light-absorbing part of the spectrum (412 nm) at a depth of 10 m, only single photons appear moving upwards (even though the sea is powered by two billion solar photons).

**Figure 5:**
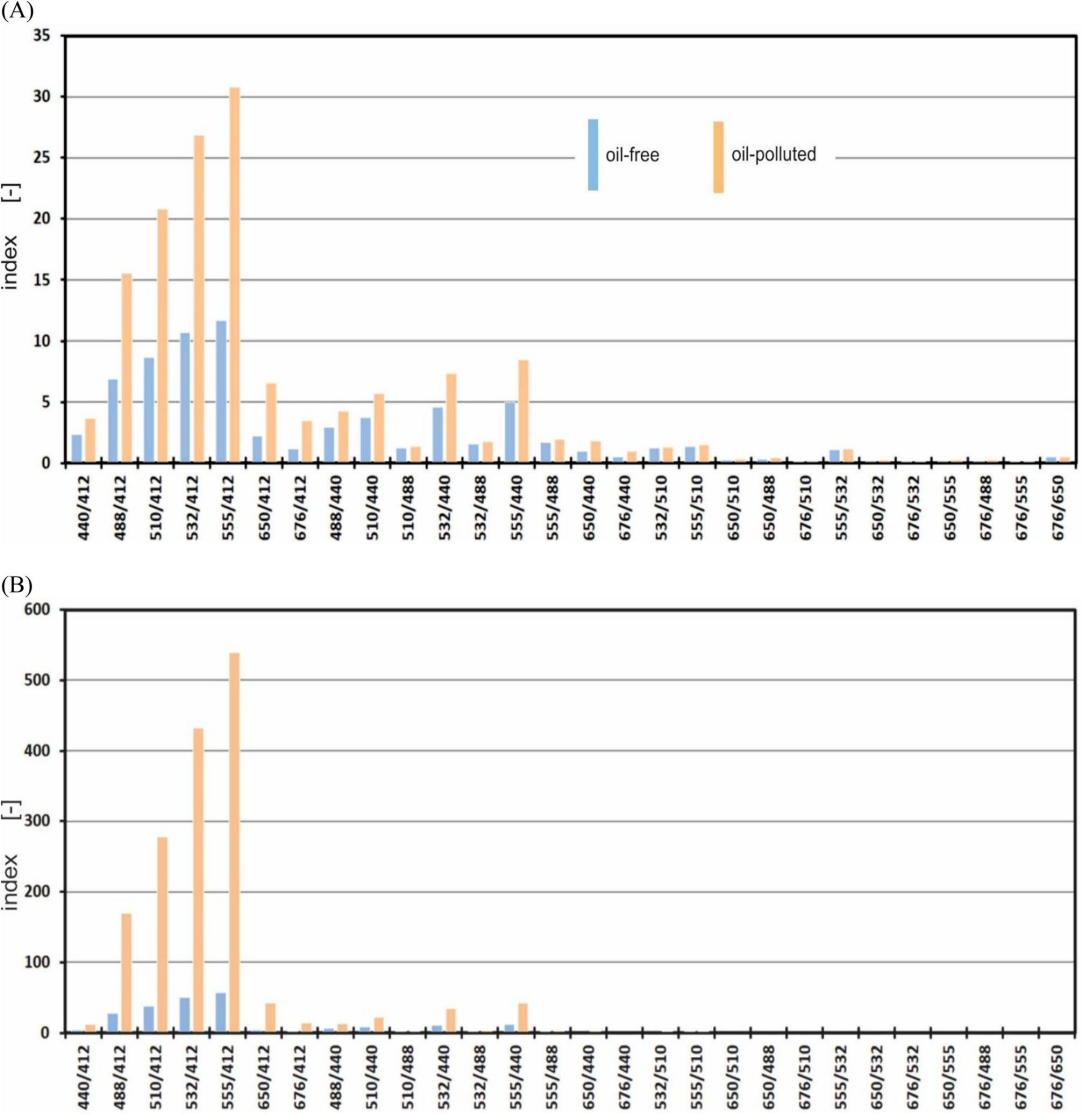
The spectral indices for both seawater free of oil (blue bars) and seawater polluted with an oil-in-water emulsion (orange bars) for readings at exemplary depths of 2 m (**A**) and 5 m (**B**).

The spectral index was defined as the ratio of the upwelling radiance for the longer wavelengths to the upwelling radiance for the shorter wavelengths. The study determines the spectral index values for all 28 combinations of wavelengths. Figure 5 shows the spectral index values for all wavelength combinations. This figure illustrates the changes in the index value for two sample depths (2 and 5 m). The nature of changes in the value of this index is similar at different depths. In each case, the optimal spectral index turns out to be I_555/412_. The index values at a depth of 5 m are about ten times higher than at 2 m.

**Figure 6:**
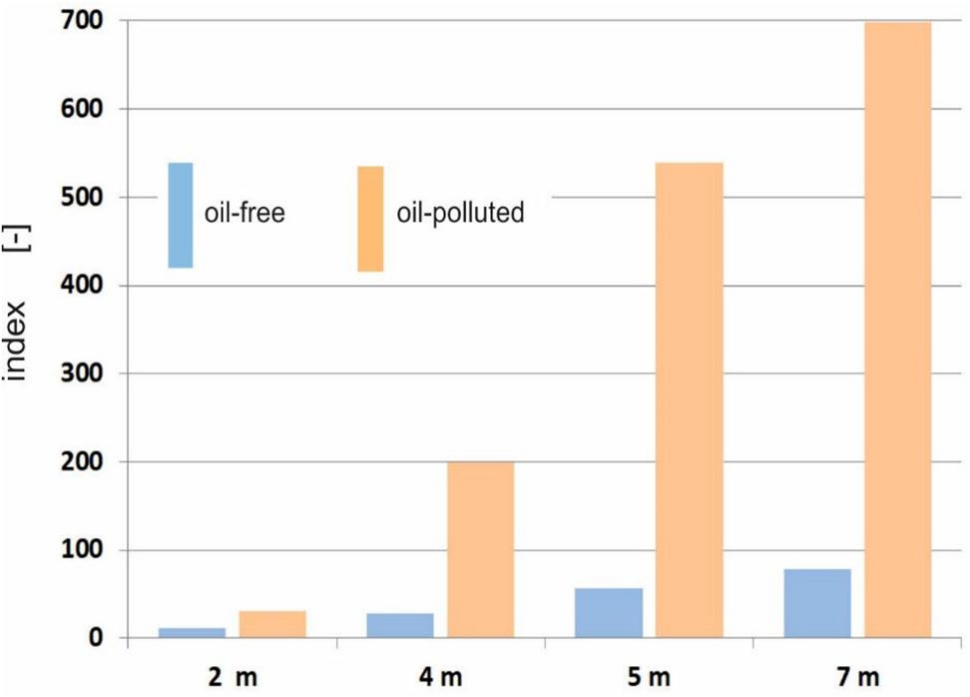
The values of the spectral index I_555/412_ determined for different depths.

The rule that the value of the spectral index increases with depth is confirmed in Fig. 6, where the values of the spectral index I_555/412_ for different depths are shown. Determining the index at a depth of 10 m turned out to be pointless because the bottom-up radiation is represented by too few virtual photons, thus making the radiation reading even more uncertain. In practice, this would require the use of a very sensitive radiance meter.

**Figure 7:**
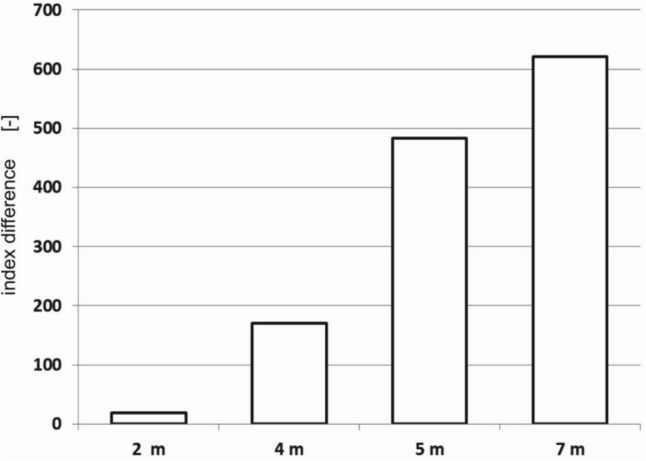
The differences between the values of the spectral index I_555/412_ for both seawater polluted with an oil-in-water emulsion and seawater free of oil.

Using the data with the values of I_555/412_ shown in Fig. 6, the differences between the index values for both clean and polluted water were determined, which are shown in Fig. 7.

The depth at which the oil sensor should be placed remains an open question because although immersing the sensor deeper would improve the efficiency of detecting dispersed oil, less and less light may not be enough to activate the sensor (Fig. 7).

**Figure 8:**
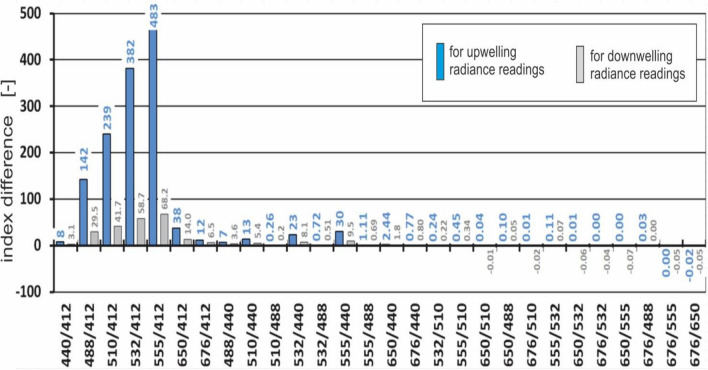
The differences between the values of the spectral index for both oil-polluted seawater and oil-free seawater (for upwelling radiance readings—received in the current paper and for downwelling radiance readings—received in the authors’ previous paper^24^).

The differences between the values of the spectral index for oil-polluted seawater and the values of the index for oil-free seawater were determined (Fig. 8).

Figure 8 shows that based on the modelling of the independent upwelling or downwelling radiance detected by a virtual underwater sensor, the most favorable combination to detect an oil emulsion in the water column is 555/412 nm. The blue bars in Fig. 8 refer to upwelling radiance readings, while the grey bars refer to downwelling radiance readings that were reported in the paper^24^. If the radiance meter is turned downwards, the difference between the 555/412 index for oil-free water and oil-polluted water increases several times. In such a situation, the radiance meter only counts the photons scattered in the water column under the detector’s location. Of course, the number of photons entering the detector also depends on the optical properties of the water above the sensor. Therefore, the presence of oil contamination in the water above the radiance sensor also affects its readings.

An additional advantage of using the upwelling radiance detector would be to avoid the settling of sediment suspensions on the sensor window.

## Conclusions

The results of analyses provided in this paper complement the information contained in the earlier paper**s**^24,31,32,37,38^, which indicate the possibility of detecting oil contaminants in the form of highly diluted oil-in-water emulsions.

The study described in the current paper was focused on analyzing the possibility of using a local optimal spectral index for a remote sensor to detect oil polluting the seawater column. For this purpose, the values of upwelling radiance for eight wavelengths under the sea surface for oil-free seawater and oil-polluted water were estimated. The obtained results concern the southern Baltic Sea. In that paper, it is suggested that the spectral index calculated using the ratio of radiation at 555 nm and radiation at 412 nm is appropriate for the detection of oily substances in the sea depths.

Moreover, the values of the spectral index (for oil-polluted seawater and oil-free seawater) based on modelling the upwelling radiance were compared with the values of the spectral index based on modelling the downwelling radiance^24^. In the light of this comparison presented in the study, the radiance meter immersed in water should be set so as to measure upwelling radiance (contrary to downwelling radiance, which was the case in papers^24,37^. The current authors’ studies followed a local optical model of the marine environment based on in situ optical measurements^29^. In semi-enclosed seas and adjacent shallow coastal waters, the correlation between optical properties and the concentration of water constituents depends strongly on specific local environmental conditions. Therefore, further research on the fundamentals of an oil sensor design based on the measurement of radiance at two wavelengths of sunlight should take into account the variability of the optical properties (IOPs) of seawater, as well as lighting (sky) and weather conditions. Semi-enclosed seas (such as the Baltic Sea), coastal areas and inland waters are among the most economically important ecosystems; unfortunately, they are also exposed to natural and anthropogenic pollution. As these areas are at risk of environmental impact from anthropogenic activities, establishing causal relationships between pollution and its consequences for these waters is fundamental^39,40^. It is expected that the local correlations established between solar light transfer and oil-in-water emulsions can be used to develop new (and improve the conventional) methods for tracking changes in ecosystems. In the future, research is planned related to the possibility of using the detection of oil dispersed in waters with various regional optical properties, in various weather conditions and in relation to possible types of oil and its concentration in water.

## Data Availability

The datasets used and analyzed during the current study are available from the corresponding author upon reasonable request.
